# QbD Supported Optimization of the Alginate-Chitosan Nanoparticles of Simvastatin in Enhancing the Anti-Proliferative Activity against Tongue Carcinoma

**DOI:** 10.3390/gels8020103

**Published:** 2022-02-09

**Authors:** Waleed Y. Rizg, N. Raghavendra Naveen, Mallesh Kurakula, Haitham A. Bukhary, Awaji Y. Safhi, Eman Alfayez, Amal M. Sindi, Sarah Ali, Samar S. Murshid, Khaled M. Hosny

**Affiliations:** 1 Department of Pharmaceutics, Faculty of Pharmacy, King Abdulaziz University, Jeddah 21589, Saudi Arabia; wrizq@kau.edu.sa (W.Y.R.); kmhomar@kau.edu.sa (K.M.H.); 2 Center of Excellence for Drug Research and Pharmaceutical Industries, King Abdulaziz University, Jeddah 21589, Saudi Arabia; 3 Department of Pharmaceutics, Sri Adichunchanagiri College of Pharmacy, Adichunchanagiri University, B.G. Nagar, Karnataka 571448, India; raghavendra.naveen@gmail.com; 4 Product Development Department, CURE Pharmaceutical, Oxnard, CA 93033, USA; 5 Department of Pharmaceutics, Collage of Pharmacy, Umm Al-Qura University, Makkah 24381, Saudi Arabia; habukhary@uqu.edu.sa; 6 Department of Pharmaceutics, Faculty of Pharmacy, Jazan University, Jazan 82817, Saudi Arabia; asafhi@jazanu.edu.sa; 7 Department of Oral Biology, Faculty of Dentistry, King Abdulaziz University, Jeddah 21589, Saudi Arabia; ealfayez@kau.edu.sa; 8 Department of Oral Diagnostic Sciences, Faculty of Dentistry, King Abdulaziz University, Jeddah 21589, Saudi Arabia; amsindi@kau.edu.sa (A.M.S.); samali@kau.edu.sa (S.A.); 9 Department of Natural Products and Alternative Medicine, Faculty of Pharmacy, King Abdulaziz University, Jeddah 21589, Saudi Arabia; samurshid@kau.edu.sa

**Keywords:** simvastatin, chitosan, alginate, central composite design, caspase-3-enzyme assay

## Abstract

The goal of the current study is to develop a chitosan alginate nanoparticle system encapsulating the model drug, simvastatin (SIM-CA-NP) using a novel polyelectrolytic complexation method. The formulation was optimized using the central composite design by considering the concentrations of chitosan and alginate at five different levels (coded as +1.414, +1, 0, −1, and −1.414) in achieving minimum particle size (PS-Y1) and maximum entrapment efficiency (EE-Y2). A total of 13 runs were formulated (as projected by the Design-Expert software) and evaluated accordingly for the selected responses. On basis of the desirability approach (D = 0.880), a formulation containing 0.258 g of chitosan and 0.353 g of alginate could fulfill the prerequisites of optimum formulation in achieving 142.56 nm of PS and 75.18% EE. Optimized formulation (O-SIM-CAN) was further evaluated for PS and EE to compare with the theoretical results, and relative error was found to be within the acceptable limits, thus confirming the accuracy of the selected design. SIM release from O-SIM-CAN was retarded significantly even beyond 96 h, due to the encapsulation in chitosan alginate carriers. The cell viability study and Caspase-3 enzyme assay showed a notable difference in contrast to that of plain SIM and control group. All these stated results confirm that the alginate-chitosan nanoparticulate system enhanced the anti-proliferative activity of SIM.

## 1. Introduction

Nanoparticles have gained huge attention as drug carriers. In particular, the polymer-based on nanoparticles [1,2], lipid-based [3,4], magnetic nanoparticles [5,6], and liposomes [7] are broadly studied under nanoformulations. Amongst them, the polymeric nanoparticles seemed to be particularly explored on account of their distinct physicochemical attributes [8]. There are natural and synthetic polymers that are flexible and are employed for many uses, inclusive of the pharma industry [9]. The polymers of natural origin are preferred more than synthetic, because of their eco-friendly cost-effective nature, biological effects, and biocompatibility.

The natural hydrogel polymers such as alginate, gelatin, and collagen have the potential to hold abundant water, keeping the structure intact, and are thus employed to carry hydrophilic drugs. The majority of the alginate produced annually is utilized for pharma and biomedical purpose, and the rest in the food industry [10,11]. Following the post-development of alginate in the 1980s, their application was expanded as microparticles for encapsulation and several studies were conducted to formulate alginate nanoparticles [12,13]. The nanomaterials of alginate denote a rapid growth field, especially in the food and pharma industry, and also in the academe [14].

Chitosan is a hydrophilic linear cationic polysaccharide found profusely in nature, consisting of glucosamine and N-acetyl-glucosamine associated with glycosidic bonds [15]. It is acquired by the removal of amino acetyl groups from chitin, a major constituent in the fungi cell wall, crustacean shells, and the cuticle of insects. Chitosan is insoluble at neutral and high pH regions due to its molecular structure and pKa (6.2–7.0). This means that chitosan can be protonated at low pH in aqueous solutions. Apart from biological degradation and biocompatibility, chitosan has a distinct bioadhesive feature that enables the interactivity of positive amino ions with a negatively charged mucous membrane. Owing to these characteristics, it is employed as a preferential matrix in the pharma industry [16].

Among the available natural polysaccharides, two polyelectrolyte polymers, chitosan and alginate having opposite charges are chosen. The advantage of the quick gelling nature of chitosan and alginate is utilized to develop polycations and polyanions of composite polyelectrolyte [17]. Employing polyelectrolytic complexation, the chitosan amine (-NH2) group and alginate carboxylic (-COOR) group interact and result in the formation of polyionic chitosan-alginate complex [18]. As this interactivity lessens the complex porosity, it can protect the encapsulant and effectually control the release than the individual chitosan or alginate [19]. At minimal pH, the high solubility of chitosan is decreased by the poor solubility of the alginate system, although at greater pH alginate is stabilized by chitosan that has low solubility.

Simvastatin (SIM) has less bioavailability (<5%) and greatly encounters metabolism by microsomal enzymes. SIM is classified as a Biopharmaceutics Classification System (BCS) Class-II compound with poor aqueous solubility and an acceptable permeability through biomembranes. The cytochrome enzyme CYP3A4 majorly targets the lactone framework of SIM and notably decreases the uptake by the intestine. The aquaphobic nature averts the drug dissolution entirely in the intestinal medium and accounts for low bioavailability. In general, statins decrease blood cholesterol by impeding HMG-CoA (3-hydroxy-3-methyl glutaryl coenzyme A) and are efficacious in maintaining cholesterol levels [20]. SIM was also recognized for its prospects in the management of various cancers by impeding the cell cycle, preventing metastasis, and promoting apoptosis [21]. Inhibition of HMG-CoA reductase results in alteration of the prenylation of small G proteins such as Ras, which regulate cell growth and survival via the downstream signaling pathways. Accordingly, inhibition of HMG-CoA reductase by statins was found to trigger apoptosis in several cancer cells. Masashi et al., recently showed that statins decreased the activation of the Ras/extracellular regulated kinase 1/2 (ERK1/2) pathway and Ras/phosphoinositol-3 kinase/Akt pathway. In malignant glioma cells, statins induce apoptosis by the activation of c-Jun N-terminal kinase 1/2 (JNK1/2) or by increasing the expression of Bim [22].

To accomplish quality by design (QbD), the strategy of Design of Experiments (DoE) is broadly employed in research and industrial setup. The conventional one factor at a time (OFAT) method for screening, development and analysis of drugs has largely been displaced by the QbD process. This novel approach renders fine results with fewer experiment trails and encompasses screening and design standardization; also it depicts the effects of various input elements and their interference in a cost-efficient mode [23]. SIM has a short half-life (about 2 h) and undergoes extensive first-pass metabolism in the intestinal gut wall and liver, thus minimizing its therapeutic efficacy. On the other side, alginate chitosan delivery systems have the potential power to improve drug stability, increase the duration of the therapeutic effect and permit administration through enteral or parenteral administration, which may prevent or minimize the drug degradation and metabolism as well as cellular efflux [24]. On basis of the above statements, the present study aims at developing alginate-chitosan nanocarriers for SIM encapsulation. Many investigators have explored the behavior of chitosan and alginate nanoparticles individually in delivering various drugs. However, SIM in chitosan nanoparticles was studied in a different manner [25], using quercetin as a doping agent. The study was not focused on cytoskeleton images and, as yet, no literature is evident on the direct use of chitosan-alginate nanoparticles as a carrier for SIM.

## 2. Results

### 2.1. FTIR (the Fourier Transform Infrared Radiation) Studies

**Figure 1:**
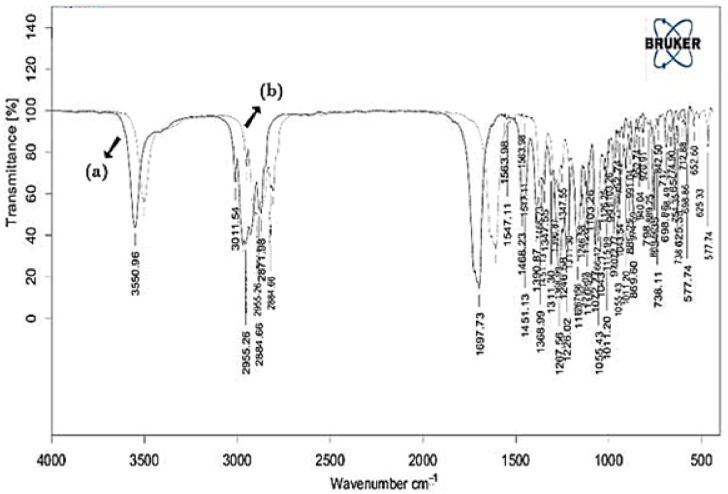
FTIR spectra of (**a**) Pure SIM and (**b**) Final formulation.

Pure SIM and final optimized formulation were studied for compatibility using FTIR and the results were demonstrated in Table 1 and Figure 1.

### 2.2. Formulation Optimization

The central composite design of response surface methodology (RSM) was employed to determine the optimum concentration of the selected factors and their interaction in the ensuing desired particle size and entrapment efficacy. A total of 13 experimental operations were projected and the responses were presented in Table 2. The particle size (PS) of all the trail preparations was observed between 109 and 351 nm, while entrapment efficiency (EE) was estimated in the range between 41 and 91%. The acquired results were examined for independent responses and the impact of parameters by statistical model fx and ANOVA. For both the responses quadratic model was used, as per the sum of squares (Type I), model summary statistics, and fit summary (Table 3 and Table 4). A quadratic high order polynomial model was chosen, where the auxiliary terms are notable and the model is not aliased.

**Figure 2:**
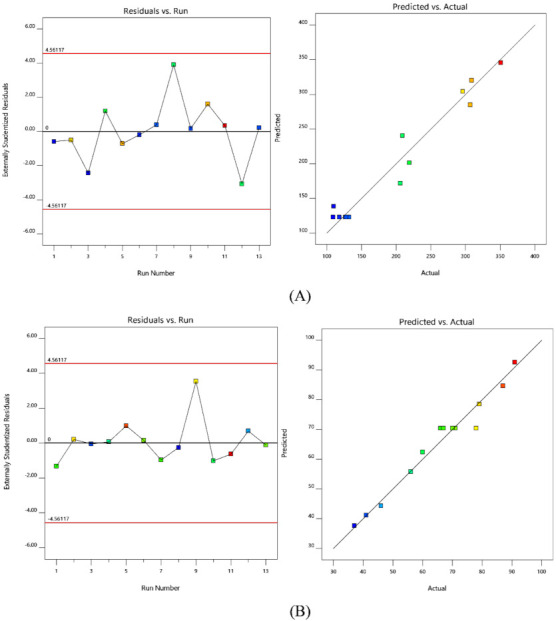
Normal probability and model residuals versus test orders for (**A**) PS and (**B**) EE.

A great interconnection was seen with the experimental and predicted values while denoting the selected responses. The probability distribution ensures that residuals are under the regular scattering, i.e., straight linearity by the points. The usual methods of statistics are not applicable, while the visible plot examination is appropriate. In addition, a general residual plot (external studentized residuals Versus usual probability percent) was employed to measure and ensure the accuracy of the adapted model [26] (Figure 2).

ANOVA was conducted to examine the intervention of quantifiable effects of the fact factors. Polynomial equations were derived by subjecting the data to multiple regressions. The equations obtained from the output of the possible optimum model were mentioned below:

PS = +122.80 − 29.56 A + 61.38 B + 21.50 AB + 60.16 A2 + 67.91 B2

EE = +70.40 − 2.33 A + 19.42 B − 0.7500 AB − 5.64 A2 − 2.64 B2

**Figure 3:**
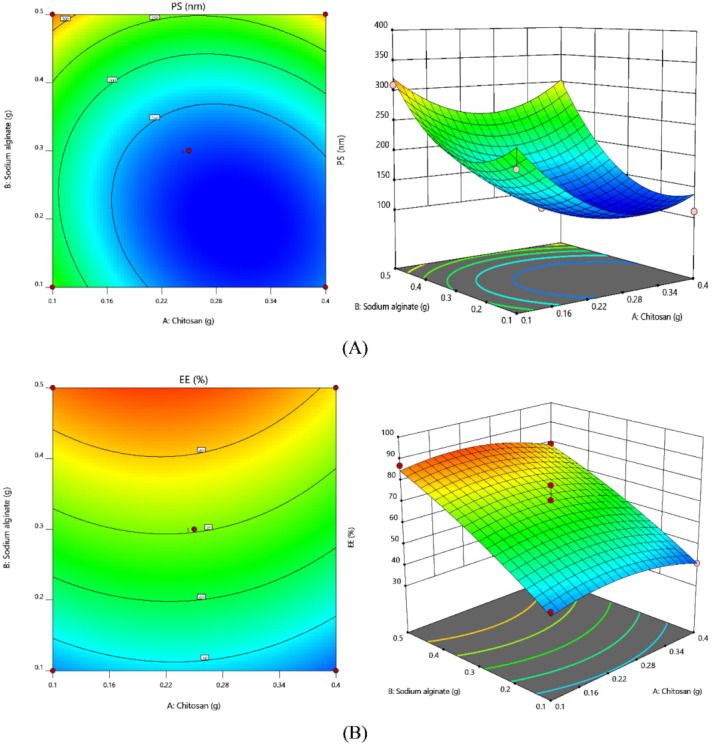
Contour plots and 3-D Response surface plots for (**A**) PS and (**B**) EE.

ANOVA coefficients with their *p* values for both the responses were shown in Table 5. Obtained results were employed to estimate the significance of model coefficients. Further, the influence of individual parameters on responses was analyzed and interpreted by RSM (Figure 3).

Global desirability function (D) was employed to standardize the model’s order obtained by statistical analysis. In the desirability function plot, the independent variables (optimal level) signified a maximum of 0.880 D value for both responses. Hence, the execution of this setting helps in achieving PS of 142.56 nm and 75.18% of EE. Relative error was calculated by comparing the predicted and experimental values and the results were shown in Table 6.

### 2.3. Zeta Potential and Polydispersity Index (PDI)

The polydispersity of standardized formulation is perhaps affirmed by a lower PDI of 0.17 ± 0.04. The zeta potential of the final formulation shows a large positive value (+36.2 mV), reflecting greater formulation stability.

### 2.4. Scanning Electron Microscopic (SEM) Studies

**Figure 4:**
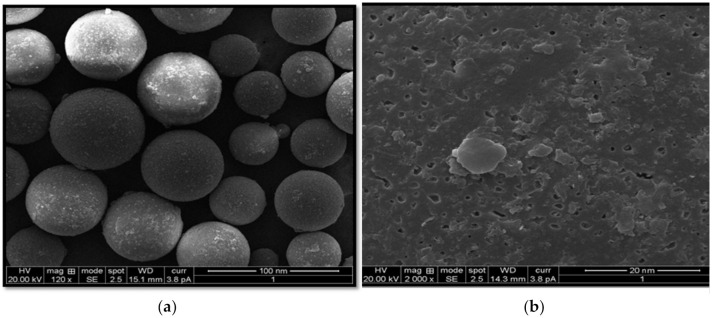
Scanning electron micrograph showing (**a**) the shape and (**b**) surface morphology of optimized SIM nanoformulation.

The surface morphology of the developed O-SIM-CAN was studied using SEM. As represented in Figure 4, spherical smooth surfaces were identified.

### 2.5. Drug Release Studies

**Figure 5:**
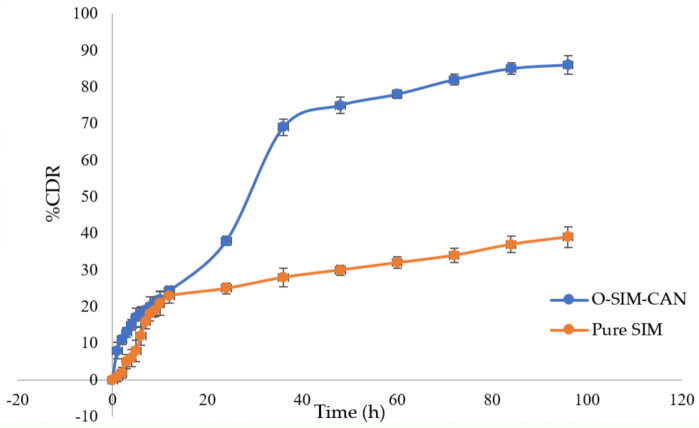
In vitro dissolution study for optimized formulation and pure SIM.

SIM release from O-SIM-CAN was determined using the dialysis membrane method and the results were presented in Figure 5. Studies were performed up to 96 h. An incomplete SIM release was observed owing to the solubility issues. Rapid release of SIM was observed for 12 h, followed by steady-state release till the end of the study.

### 2.6. Cell Viability Studies

**Figure 6:**
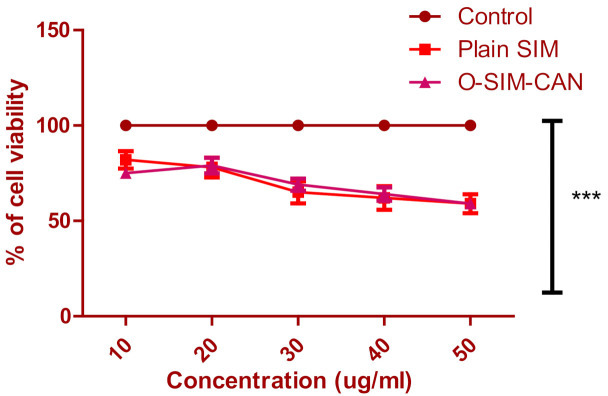
O-SIM-CAN, plain SIM, and control [5 Fluorouracil] effect on the percentage cell viability of HSC-3 cell lines. (The values indicated were the Mean ± S.D, n = 9).

**Figure 7:**
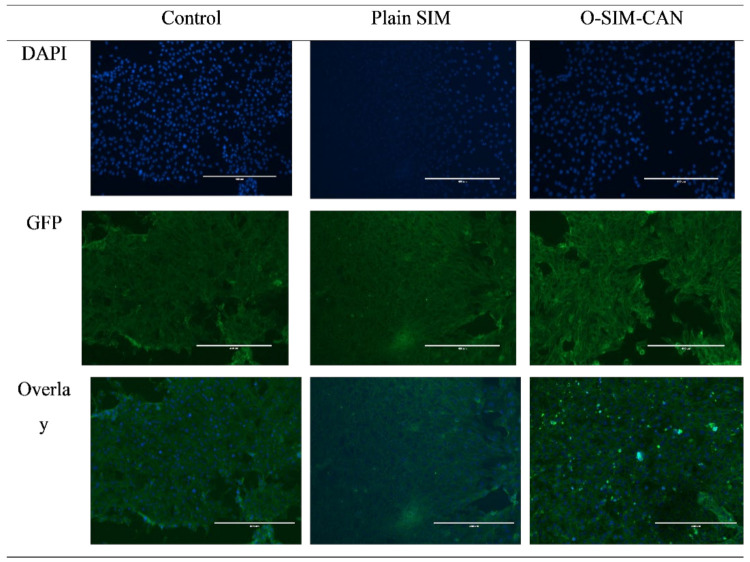
Cytoskeleton images of control, SIM, and O-SIM-CAN after 24 h treatment.

**Figure 8:**
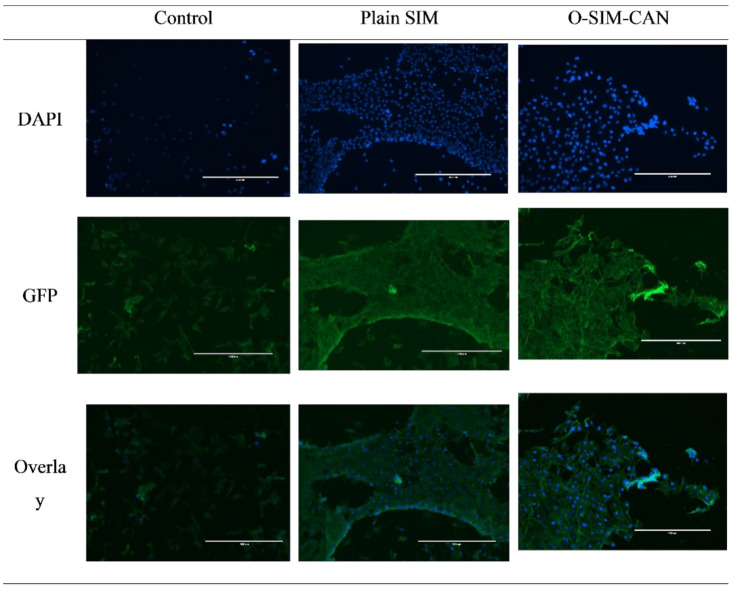
Cytoskeleton images of control, SIM, and O-SIM-CAN after 72 h treatment.

MTT assay was employed to determine cytotoxicity over HCS-3 cells for the optimized formulation of SIM and plain SIM following 72 h of treatment. Figure 6 shows the differentiation of cell viability percent of the preparations with the control group (normalized to 100). At initial concentration, the cell viability of SIM was found to be 82 ± 4.6% and at higher concertation, it declined to 59 ± 4.6%. A similar pattern was observed with optimized formulation. Cytoskeleton images of control, SIM, and O-SIM-CAN after 24 h and 72 h treatment were shown in Figure 7 and Figure 8. 

### 2.7. Caspase-3 Enzyme Assay

**Figure 9:**
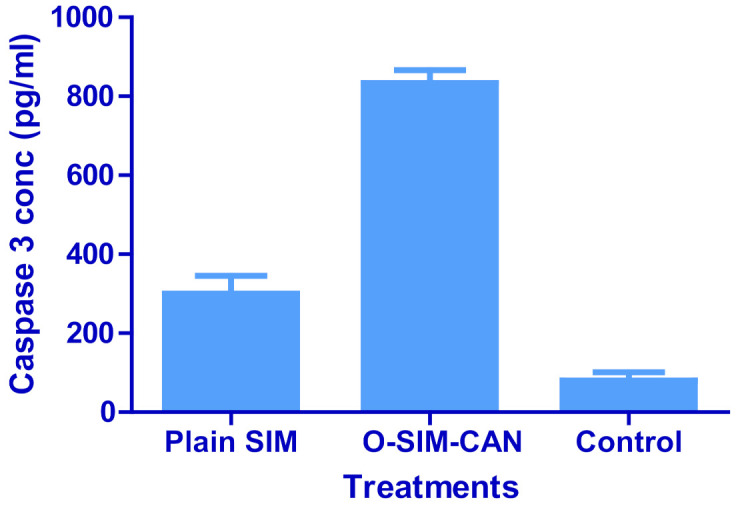
Concentrations of enzyme caspase-3 in HCS 3 cells treated with plain SIM, O-SIM-CAN, and control (solvent-free); (n = 6 ± SD).

The test was conducted as per the specifications by introducing the samples in 96 well plates. Notable caspase-3 levels were detected in HCS-3 cells treated with the optimized formulation (834.25 ± 32.84 pg/mL), as shown in Figure 9. In contrast to control, SIM showed a considerable rise in the concentration (300.25 ± 45.18 pg/mL). 

## 3. Discussion

IR Spectrum of the pure SIM shows the characteristic peaks at 3550, 2931, 1465, and 1072 cm^−1^. From the IR spectral data of SIM formulation, it is evident that there were no interactions of the drug as it exhibited similar peaks with a slight change in the intensity. This confirms the undisturbed structure of the drug in the formulation. Thus, this proves the fact that there is no potential incompatibility of the drug with the chosen polymers for formulation.

The root effect interrelationship among selected variables and the independent responses could be demonstrated by the recommended quadratic polynomials and the corresponding statistical significance, determined by ANOVA. The Predicted R² for both the responses of 0.891, 0.9223 is in accordance with the Adjusted R² of 0.9209 and 0.9463, respectively, as the variation falls below 0.2. In addition to this fit, summary data were applied to ensure the effectiveness and fitness of the chosen model. The model repeatability can be assured with the value of the coefficient of variation (CV). CV of the selected quadratic model should be <10%, to confirm the reproducibility. Relatively low CV values (7.35-PS & 5.98-EE) were noted in the study which ensures model accuracy and reliability. Adequate Precision quantifies S/N (signal to noise) proportion. In general, a fraction greater than four is preferable. PS and EE show a ratio of 13.1592 and 20.6875, denoting an appropriate signal, thus confirming the efficiency of the model to run the design space. Lack of fit can result in an ineffective model to represent the complete data. Therefore, lack of fit is a prerequisite to determining that the equations developed by the model are coherent in forecasting the responses. The lack of fit *p* values of PS, EE, and SI were observed as insignificant and so the model chosen was appropriate [27]. The Model F-value of both the responses were found to be 28.92 and 43.33, inferring the applicability of the model. Only a probability of 0.01% exists that a large F-value may arise because of the noise and as required, the model *p*-value was observed to be significant with *p* values of 0.0002 and <0.0001.

Further, the effect of test orders on the adapted model was illustrated by the residuals versus test order [28]. In the present work, linear distribution of the external studentized residuals with a slight variation was noted, denoting that the selected model was admissible statistically [29]. Figure 1b, depicts experimental operations set against the residuals, indeed a working method to recognize the slinking variables which may alter the study results. An arbitrary distribution pattern is noted in the chart that denotes time-dependent variables lurking in the framework.

ANOVA results outranged the statistical significance developed by the quadratic equation; furthermore, the *p*-value was <0.0500, representing the significance of model terms. The test method stipulated that PS was greatly influenced by (a) adversary effect of A with *p*-value 0.0121 and (b) synergic effects of B, and polynomial terms of A and B with a *p*-value of 0.0002, 0.0004, and 0.0002, correspondingly. Response 2 was greatly impacted by (i) adversary effect of polynomial term of A with *p*-value of 0.0067 and (ii) synergism effect of B with a *p*-value of <0.0001, and amongst the crucial parameters, term B affected the EE with high enormity.

The contour plot which gives the association of chosen responses with the variables ensures the variable effects. RSM was employed to estimate and interpret the response of independent parameters against the obtained discrete responses. Three-dimensional surface graphs are crucial to illustrate the interactivity and main effect. The obtained responses are forecasted by contour plots [30]. As seen in contour plots, PS was found to be less as the chitosan concentration increased; in contrast to this, a high concentration of sodium alginate will be responsible for higher particle sizes. The interaction of these variables (blue color region) at specified concentration can yield the nanoparticles with minimum PS. Maximum EE was observed (orange color zone) with a high concentration of sodium alginate and varied concentration of chitosan. All these results will comply with ANOVA and regression analysis.

To standardize the model’s order obtained from statistical analysis, the function of global desirability (D) was employed. Every response was laid a limit (PS-Minimum and EE-Maximum) to draft an inlay plot to enhance the independent variables. All the feasible individual parameters were included in the method for standardization. The optimized concentrations of chitosan and sodium alginate were found to be 0.258 g and 0.353 g with desirability of 0.880. An optimized formulation (O-SIM-CAN) was prepared and evaluated for PS and EE to validate the study design. As required, the relative error was observed below 5%, which confirms the design accuracy (Table 6). The same formulation was used to evaluate the remaining parameters.

Initially, in the preparation of SIM-CA-NP chitosan droplets were formed while stirring with tween 80. Subsequently, solidification was observed because of ionic crosslinking with alginate solution. Chitosan acquired a positive group, owing to the presence of amine groups at the aqueous solution of pH 4 to 5.5, while alginate dissolved in a neutral pH solution where the carboxylic groups were charged negatively. Hydrogel is formed due to the interactivity of amino groups of chitosan and carboxylic groups of alginates in the aqueous solutions of nearly 5.2 pH. At the same time, SIM—which is positively—charged was complexed with negatively charged alginate to attain a greater drug loading to the nanoparticle. The preparation rendered an opalescent suspension with a positive value of zeta potential. Chitosan acquired a positive group, owing to the presence of amine groups at the aqueous solution of pH 4 to 5.5, while alginate dissolved in a neutral pH solution where the carboxylic groups were charged negatively. Hydrogel is formed due to the interactivity of amino groups of chitosan and carboxylic groups of alginates in the aqueous solutions of nearly 5.2 pH. At the same time, SIM—which is positively charged—was complexed with negatively charged alginate to attain a greater drug loading to the nanoparticle. The preparation rendered an opalescent suspension with a positive value of zeta potential. Final NPs were collected after freeze-drying. The high positive surface charge of the formulation is an additional benefit while employing NPs in drug delivery as they can be easily transported by the negative channels in the plasma membrane. The variation in the zeta potential is attributed to the neutralization of the chitosan charge by the powerful negative charge of STTP versus alginate at the working pH. The standardized PDI validates the monodispersity of the formulation. Various structures of smooth to rough structures were noticed, and these may likely be developed by the formation of the hydrogen bond between eNH2, eOH, and eNHCOCH2 groups of chitosan backbone. Further SEM images clearly showed the well-separated and disperse nanoparticles. In addition to this, the SEM image gave a rough estimation regarding the particle size, which was about 20–40 nm in contrast to particle size determination by the DLS method. This can be explained since DLS measures the hydrodynamic diameter and also the swelling capability of polymeric hydrogel in the solution, while SEM depicts the pictures of dried particles.

The cumulative SIM release from plain SIM and the optimized formulation was studied as a function of time using PBS solution of pH 7.4. In the beginning, a quick release of SIM from both plain SIM and O-SIM-CAN is seen up to 24 h. It contributes around 40–45% of SIM from the total encapsulated quantity. This initial rapid release of SIM from NPs was mainly attributed to the occurrence of SIM at the NP’s surface, allowing a great extent of water diffusion through the liquid matrix, and thus accounts for rapid drug release. Further, a sustained phase with consistent drug release is seen for the next 72 h. The two profiles had a similar pattern of release, yet variation exists in the quantity released. The total amount of drugs released from the optimized formulation was around 86.25%. This was because of the gelling action of chitosan and alginate, which were responsible for controlling the drug release. On the contrary, the amount of total drug released from plain SIM was nearly 35%. It certainly denotes that the release of SIM was decelerated due to the encapsulation of NPs.

Two treatments (plain SIM, optimized formulation) at distinct concentrations (10–50 µg/mL) and the control group were tested for cell viability. This test affirms that all the treatments decreased cell viability with the given dose (in a dose-dependent manner). The observed percentage cell viability of O-SIM-CAN was less than the plain SIM, denoting that the effect resulted in high cytotoxicity on HSC-3 cells. The cytoskeleton images indicate that O-SIM-CAN showed uniform distribution and extreme cellular spreading. This nature was observed the same for 24 h and 72 h of cell culture in contrast to other samples. This can be due to, the surface nature of the chitosan alginate nanoparticles, which further promotes the strong affinity to the HSC-3 cells on a porous surface. Cell migration and vascularization were further noticed. That leads to differentiation and proliferation of the cells for the new tissue growth. In the case of pure SIM, this nature of differentiation was observed to be poor, owing to its inability towards the HSC-3 cells. The slightly augmented property was observed after 72 h of cell treatment. Hence, the chitosan alginate carrier system assisted in improved cell proliferation for SIM [31]. Literature suggests that chitosan and its derivatives can selectively pervade through the cancer cells and exhibit antineoplastic effects employing cellular enzymes, apoptosis, antiangiogenic, enhanced immunity, and antioxidant defense mechanisms. On the other hand, alginate-based carriers modified with several drugs are presumed to accumulate in the liver and have a high level of targeting efficiency to hepatocytes. Hence, both these ingredients were majorly responsible for the enhancement of the anti-proliferative activity of SIM. The caspase-3 enzyme assay and cell viability studies ensure that the occurrence of apoptosis with optimized formulation increased significantly compared to other treatments, which can be credited to the use of chitosan and alginate in designing the current formulation.

## 4. Materials and Methods

### 4.1. Materials

Simvastatin was generously presented by Biocon Pvt.Ltd, Bangalore, India. Sodium alginate and chitosan (average molecular mass, deacetylation level ∼75%) were procured from Sigma Aldrich, St. Louis, MO, USA. Ethanoic acid and Tween 80 were purchased from Acros organics, Morris Plains, NJ, USA. The National Centre for Cell Sciences (NCCS), India, presented the HCS-3 cell lines. All other reagents and chemicals employed were of analytical purity grade and procured from standard manufacturers.

### 4.2. Methods

#### 4.2.1. FTIR Studies

FTIR spectral measurements were taken at ambient temperature using an IR spectrophotometer (Perkin Elmer Instruments, Waltham, MA, USA). These were completed qualitatively to assess the pattern of peaks and for comparison purposes [25]. The FTIR spectra pure SIM and final formulation were taken by making a KBr disc and analyzed in the range of 400–4000 cm^−1^ [32].

#### 4.2.2. Preparation of SIM Loaded CA-NP

Preparation of SIM Loaded Chitosan-Alginate Nanoparticles (SIM-CA-NP) was completed by a polyelectrolytic complexation technique. The chitosan flakes were dispensed in 10 mL of ethanoic acid (0.3% *v*/*v*) and the obtained solution pH was adjusted to 4.8. The alginate solution was prepared by using 100 mL purified water at ambient temperature overnight and the pH was adjusted to 5.2. The alginate solution and SIM (5 mg) were agitated continuously for 24 h to allow the formation of the drug alginate complex. The prepared solution of chitosan and Tween 80 (0.310 g) were mixed and agitated at 60 °C for 120 min to obtain a uniform mixture and slowly this solution was dribbled to the alginate-SIM complex for 60 min while agitating at a high rate [33]. The stirring was continued for another 30 min and then freeze all night. Then a nanoparticle suspension was centrifuged at 9000 rpm for 45min to dry the obtained pellet in a vacuum overnight to obtain the nanoparticles after drying. Then a nanoparticle suspension was centrifuged at 9000 rpm for 45min to obtain the nanoparticles after drying.

#### 4.2.3. Optimization of SIM-CA-NP Preparation

The preparation of SIM-CA-NP was standardized using RSM and various statistical applications. This methodology aids to determine (i) ideal process conditions, (ii) notable factors and their association by fewer experiment operations. The process parameters chosen were, chitosan concentration (X1), and sodium alginate concertation (X2) at five levels encoded as −1.414, −1, 0, +1, and +1.141. These parameters were optimized for PS (Y1) and EE (Y2). Design Expert V.12 was employed to apply the central composite model, that can develop 13 investigation trails. Table 7 exhibits the total work plan interns of coded and real values of parameters chosen and limitations of dependent factors. The developed polynomial equations were validated statistically using ANOVA. Entire trail runs were subjected to distinct statistical models such as 2FI, quadratic and compared based on various parameters such as relative standard deviation, multiple correlation coefficient (R2), predicted, adjusted R2 values, and the ideal model was chosen [34]. Quadratic regression was applied to quantify the response in each experiment and an investigation was conducted.

#### 4.2.4. Characterization

##### Estimation of PS and Polydispersity Index (PDI)

SIM-QRC NPs were examined by Zetasizer (Malvern Instruments, Malvern, UK) to determine particle size and dispersity index through the light scattering method [35]. Using deionized water (10 mL), samples were made by diluting an adequate amount of formulation. The evaluation was undertaken at 25 °C with refractive index-1.33; He-Ne Red laser, 4.0 mW at 633 nm, and every quantification was undertaken thrice.

##### EE

EE is described as the proportion of total drugs amount observed in the formulation and it was conducted the indirect way. The developed SIM-CA-NPs were taken in a petri dish and subjected to freeze-drying and a further adequate quantity of acetonitrile was put in and agitated rigorously [36]. Centrifugation was completed for 60 min at a rate of 10,000 rpm and the supernatant fluid was collected. In addition, cleaning was performed using acetonitrile and all the washings were obtained. Supernatant fluid and the cleaning contents were combined and drained with a water bath and the resultant was diluted by methyl alcohol. The absorbency of SIM was measured at 450 nm. EE was computed based on the formula below;

$$EE\left(\%\right)=\frac{C_{total}-C_{free\;}}{C_{total}}\times 100$$

where,

*C_total_* is the theoretical amount.

*C_free_* is the quantity of drug found in the supernatant.

#### 4.2.5. Standardization and Validation of Optimization Outcome

Design-Expert software was employed to instigate the responses provided by all the preparations. The responses were utilized to develop the study methodology and the response surface graph. A numerical standardization method was employed to develop an optimized formula with a specified minimal and maximal limit of every parameter. The results were integrated into a desirability function. The set of solutions was classified with the highest desirability and the solutions which met the specifications are noted. The relation of the independent and dependent parameters was elucidated by the response surface graph. The influence of various factors on the slope coefficients was studied by ANOVA. As a part of design validation, the relative uncertainty was enumerated using the dissimilarity of predicted and experimental values.

#### 4.2.6. The Rationale of Experimental Design

An optimized formulation of SIM (O-SIM-CAN) was prepared by utilizing the independent variables in optimum concentrations as specified by software and assessed. The standardized results of the test design can be substantiated by computing an absolute error using the predicted and practical responses as shown in the following equation,

$$\mathrm{Relative}\;\mathrm{error}\left(\%\right)=\frac{\mathrm{Predicted}\;\mathrm{value}-\mathrm{Practical}\;\mathrm{value}}{\mathrm{Predicted}\;\mathrm{value}}$$

#### 4.2.7. PDI and Zeta Potential Determination

The sample was diluted with distilled water (1:10) in the capillary cell at 25 °C. Both PDI and surface charge were measured for nano complex formulations using Malvern Zetasizer (2000, Malvern, UK) [37].

#### 4.2.8. SEM

To examine the surface structure and configuration of the developed nanocomposites SEM was utilized. The sample was placed on a stub and sputter-coated with gold and studied using SEM (JEOL, JSM-6100, Tokyo, Japan).

#### 4.2.9. Drug Release Studies

SIM release form optimized formulation was studied in distilled water and phosphate-buffered saline (PBS) pH 7.4 under sink conditions. The pellet of separated nanoparticles was dispersed in 5mL PBS (7.4 pH) entrapped in a dialyzer membrane (with 3.5 kDa molecular weight cut-off) and submerged in 25 mL PBS. This setting was maintained at 37 °C with gentle stirring. At the pre-established periods, aliquots of 3 mL were drawn, and absorbance of the sample was recorded. Fresh medium was replaced following each withdrawal. The study was replicated thrice, and the mean data were noted.

#### 4.2.10. In Vitro Cell Viability Assay

The cytotoxicity of standardized formulation (O-SIM-CAN) was determined by the in-vitro model over HCS-3 cells by MTT assay (3-[4,5-dimethylthiazol-2-yl]-2,5-diphenyltetrazolium bromide). HCS-3 cells with 5 × 10^4^ cells/mL density were subjected to trypsinization using 0.25% trypsin-EDTA and placed in 96-well plate (nearly 2500–5000 cells/well). Subsequently, after 24 h, the entire medium was displaced with DMEM (Dulbecco’s Modified Eagle Medium). The reference cells were served with 5-fluorouracil and the other cells are given with absolute test samples at a range of 10–50 μg/mL. Following the incubation period of 72 h, DMEM of 0.1 mL containing 0.2 mg/mL MTT was added and incubated for 2–3 h. To dismiss the formed formazan, DMEM was substituted with DMSO. Later the absorbance was determined by a microplate reader (Biotek Synergy, Santa Clara, CA, USA) at 540 nm. Finally, the dose-response relationship was established and IC 50 values were estimated [25].

Additionally, the cytoskeletal structure was studied using an inverted microscope to confirm cell viability. Around 500 µL of 3.7% formaldehyde (in MES-N-morpholino ethane sulfonic acid) was further added to each well, having tissue culture plastic (control cells) and a test sample. These cells were incubated for 10 min and then formaldehyde solution was removed, after washing with phosphate buffer solution. To permeabilize the cells for staining, 500 µL of 0.5% TritonX100 in MES buffer was added and leftover for 305 min. Leftover TritonX100 was removed, and the samples were again washed with phosphate buffer. Alexa Fluor 488 (200 µL) stained each sample and they were incubated for 20 min. While incubating, samples were covered and protected from light. After incubation, cells were separated from the wells using forceps and then invert on a large glass slide. An inverted microscope (Nikon Eclipse TE300, Nikon, Tokyo, Japan) with a mercury lamp light source was used for the examination [38]. DAPI (4,6-diamino-2-pheylindole) was given DNA blue fluorescence and Green fluorescent protein (GFP) and finally, both DAPI and GFP were recorded.

#### 4.2.11. Caspase-3 Enzyme Assay

The caspase-3 enzyme test was conducted for the control group, O-SIM-CAN, and plain SIM. The cells were first grown in ROMI 1640 (consisting of 10% fetal bovine serum at 37 °C) and cell lysis buffer was employed to break down the cell. The resultant lysate was collected and diluted with the classic buffer in accordance with the assay specifications for HCS-3 traces and the cells were overlaid in DMEM (100 μL) at 1.3–1.9 × 10,000 cells/well. One day before the caspase assay, each sample was inoculated in a 96-well plate [39]. The assay was performed (in line with the kit instructions-USCN Life Science Inc., Wuhan, China) by Spectrophotometry at 450 nm.

## 5. Conclusions

In the current study, the low soluble drug simvastatin was effectively loaded into chitosan alginate nanoparticles. The QbD based models were applied along with the overall desirability in standardizing the preparation of SIM-CA-NP. As per the desirability approach, a formulation containing 0.258 g of chitosan and 0.353 g of alginate can fulfill the requirements of optimum formulation for preparing O-SIM-CAN. Relative error was found to be within the acceptable limits, thus confirming the accuracy of the design. Higher positive zeta potential indicates the high stability of the formulation. Relatively low PDI and SEM studies further confirm the monodispersity and surface characteristics of the formulation. The controlled release of SIM from optimized formulation can be presumed to encourage or assist in angiogenesis. Prepared nanoparticle formulation exhibited a notable improvement in apoptosis mediated by caspase-3 and elevated the levels of tumor suppressor protein. Thus, the overall attributes of SIM–CA-NP make it a novel formulation that can create a new pathway to treat carcinomas.

## Figures and Tables

**Table 1:** Interpretation of FTIR spectra of pure SIM and formulation.

S. No	Wavenumber Identified in SIM (cm^−1^)	Functional Group Assigned	Wavenumber Identified in Formulation (cm^−1^)
1.	3550	O-H Stretch	3559
2.	2931	C-H stretch: Methyl/methylene asymmetric	2942
3.	1465	Methylene symmetric C-H bend	1472
4.	1267	-C-O-C bend (Lactone)	1271
5.	1165	-C-O-C bend (Ester)	1169
6.	1072	C-O Stretch (secondary alcohol)	1073
7.	1566	N-H stretch	1569

**Table 2:** Projected experimental runs for central composite design and their observed responses.

		Factor 1	Factor 2	Response 1	Response 2
Std	Run	A: Chitosan	B: Sodium Alginate	PS	EE
		(g)	(g)	(nm)	(%)
10	1	0.25	0.3	109	66
4	2	0.4	0.5	296	79
2	3	0.4	0.1	110	41
6	4	0.462132	0.3	219	56
3	5	0.1	0.5	309	87
11	6	0.25	0.3	118	71
12	7	0.25	0.3	132	67
7	8	0.25	0.0171573	206	37
9	9	0.25	0.3	127	78
5	10	0.037868	0.3	307	60
8	11	0.25	0.582843	351	91
1	12	0.1	0.1	209	46
13	13	0.25	0.3	128	70

**Table 3:** Model Summary Statistics of selected responses.

Responses	Source	Sequential *p*-Value	Lack of Fit *p*-Value	Adjusted R²	Predicted R²	
Lag Time	Linear	0.0811	0.0002	0.2738	0.0095	
	2FI	0.5958	0.0002	0.2194	−0.0863	
	Quadratic	0.0001	0.2114	0.9209	0.8919	Suggested
	Cubic	0.5849	0.0037	0.9106	−1.1565	Aliased
T- 95% CDR	Linear	<0.0001	0.2628	0.8750	0.8363	
	2FI	0.8162	0.2116	0.8620	0.8172	
	Quadratic	0.0152	0.8480	0.9463	0.9223	Suggested
	Cubic	0.8315	0.5374	0.9302	0.7696	

**Table 4:** Model (Quadratic) fit summary of the responses.

Parameter	PS	EE
Std. Dev.	24.90	3.91
Mean	201.62	65.31
C.V. %	7.35	5.98
Adeq Precision	13.1592	20.6875
Lack of Fit F-value	15.55	12.2650
Lack of Fit *p*-value	0.0614	0.8480
Model F value	28.92	43.33
Model *p* value	0.0002	<0.0001

**Table 5:** ANOVA coefficients table for both the responses.

	Intercept	A	B	AB	A²	B²
PS	122.8	−29.5563	61.3826	21.5	60.1625	67.9125
*p*-values		0.0121	0.0002	0.1279	0.0004	0.0002
EE	70.4	−2.33211	19.4209	−0.75	−5.6375	−2.6375
*p*-values		0.1353	<0.0001	0.7125	0.0067	0.1183

**Table 6:** Relative error calculation for optimized formulation.

S. No	Response	Predicted/Theoretical Value	Experimental/Practical Value	Relative Error (%)	Limit for Relative Error (%)
1.	PS (nm)	142.56	143.23	−0.47	±5
2.	EE (%)	75.18	74.72	0.61	

**Table 7:** Entire work plan interns of coded and real values of parameters chosen and limitations of dependent factors for central composite design.

Selected Formulation Factors	Levels					Responses/Dependent Variables	Constraints
	−1.141	+1	0	+1	+1.141		
Chitosan (g)-X_1_	0.037	0.1	0.25	0.4	0.462	Particle size (nm)	Minimum
Eudragit RLPO (g)-X_2_	0.017	0.1	0.3	0.5	0.582	EE (%)	Maximum

## Data Availability

All data available are reported in the article.
